# Usefulness of MCP-1 Chemokine in the Monitoring of Patients with Coronary Artery Disease Subjected to Intensive Dietary Intervention: A Pilot Study

**DOI:** 10.3390/nu13093047

**Published:** 2021-08-30

**Authors:** Magdalena Makarewicz-Wujec, Jan Henzel, Cezary Kępka, Mariusz Kruk, Łukasz Wardziak, Piotr Trochimiuk, Andrzej Parzonko, Zofia Dzielińska, Marcin Demkow, Małgorzata Kozłowska-Wojciechowska

**Affiliations:** 1 Department of Clinical Pharmacy and Pharmaceutical Care, Medical University of Warsaw, 02-091 Warsaw, Poland; mkw@wum.edu.pl; 2 Department of Coronary and Structural Heart Diseases, Cardinal Stefan Wyszynski Institute of Cardiology, 04-628 Warsaw, Poland; ckepka@ikard.pl (C.K.); mkruk@ikard.pl (M.K.); lukas.wardziak@gmail.com (Ł.W.); ptrochimiuk@ikard.pl (P.T.); zdzielinska@ikard.pl (Z.D.); mdemkow@ikard.pl (M.D.); 3 Department of Pharmacognosy and Molecular Basis of Phytotherapy, Medical University of Warsaw, 02-091 Warsaw, Poland; andrzej.parzonko@wum.edu.pl

**Keywords:** DASH diet, chemokine MCP-1, atherosclerotic plaque

## Abstract

Monocyte chemotactic protein-1 (MCP-1) plays an important role in the entire atherosclerotic process, from atherogenesis to destabilisation of the atherosclerotic plaque. The purpose of this study is to evaluate the effect of the dietary approaches to stop hypertension (DASH) diet in patients with coronary artery disease on the MCP-1 plasma concentration and to evaluate the potential usefulness of this chemokine as a marker of change in the volume and composition of coronary plaque. Material and method. As part of the dietary intervention to stop coronary atherosclerosis in computed tomography (DISCO-CT) study, patients were randomised to an intervention group (*n* = 40) in which the DASH diet was introduced, and to a control group (*n* = 39) with no dietary intervention. In the DASH group, dietary counselling was provided at all follow-up visits within 12 months of the follow-up period. MCP-1 plasma concentration was determined using enzyme-linked immunosorbent assay (ELISA). Coronary plaque analysis was performed using a semi-automated plaque analysis software system (QAngioCT, Medis, The Netherlands). Results. In the DASH group, MCP-1 plasma concentration significantly decreased by 34.1 pg/mL (*p* = 0.01), while in the control group, the change in MPC-1 was not significant. Significant inverse correlations were revealed for the change in MCP-1 plasma concentration and change in the consumption of vitamin C and dietary fibre both in the DASH (r = −0.519, *p* = 0.0005; r = −0.353, *p* = 0.025, respectively) and in the control group (r = −0.488 *p* = 0.001; r = −0.502, *p* = 0.001, respectively). In patients with the highest decrease in percent atheroma volume (PAV), a significant positive correlation was observed between the change in MCP-1 plasma concentration and changes in PAV (r = 0.428, *p* = 0.033) and calcified plaque component (r = 0.468, *p* = 0.018), while the change in noncalcified plaque component correlated inversely with change in MCP1 (r = −0.459, *p* = 0.021). Conclusion. Dietary intervention based on the DASH diet model reduces the MCP-1plasma concentration, mostly due to an increased intake of plant-derived, fibre-rich foods and antioxidants. The change in MCP-1 plasma concentration seems to reflect changes in the atheroma volume and proportions between the calcified and non-calcified plaque elements.

## 1. Introduction

Inflammation is a central process in the development and destabilisation of atherosclerotic plaques, leading to serious cardiovascular events such as myocardial infarction and stroke [1,2,3]. A number of chemokines is involved in the complex process of atherosclerotic plaque formation to recruit of various leukocytes, such as monocytes, macrophages and T lymphocytes, to the site of developing atheroma [4]. Monocyte chemotactic protein-1 (MCP-1), also known as C-C motif chemokine ligand 2 (CCL2), is primarily secreted by inflammatory and endothelial cells, triggering the recruitment of monocytes to the inflammation site [5,6]. Numerous studies have shown that MCP-1 plays an important role in the entire atherosclerotic process, from atherogenesis to destabilisation of the atherosclerotic plaque [7]. Higher MCP-1 concentrations in the atherosclerotic lesions were shown to be associated with histopathologic markers of plaque vulnerability [8]. Similarly, circulating forms of MCP-1 are found in plasma, and their concentration increases in the presence of active inflammation [9]. Studies indicate that a higher level of circulating MCP-1 is related to a higher stage, phenotype and vulnerability of atherosclerotic plaque [10]. Moreover, MCP-1 has been indicated as an independent diagnostic marker in subjects with a history of acute coronary syndrome since higher MCP-1 levels were related to a higher risk of death, myocardial infarction and heart failure [11,12]. A meta-analysis of seven cohort studies indicates that higher MCP-1 levels are related to a higher long-term cardiovascular mortality also in subjects with no evident cardiovascular disease (CVD), [13]. Other studies confirm these observations, suggesting the usefulness of MCP-1, not only as a risk biomarker, but also as a potential therapeutic target in inflammatory and atherosclerotic diseases [14,15]. It has been shown that statins have a significant anti-inflammatory and antiatherogenic effect, at least partially through MCP-1 inhibition [16,17,18].

There are few reports on the effect of dietary intervention on the MCP-1 plasma concentration. The long-term use of a Mediterranean diet has been shown to significantly reduce MCP-1 concentration the level of this chemokine in subjects with confirmed atherosclerotic lesions [19]. Thus far, there are no such studies for the dietary approaches to stop hypertension (DASH) diet which, aside to the Mediterranean diet, is the most widely used dietary model in the treatment and prevention of CVD [20]. The DASH diet provides primarily plant-based foods and low-fat dairy products and is poor in animal-based foods (especially those high in saturated fatty acids and cholesterol) and foods with a high glycaemic index [21]. In previous studies, we showed that an intervention based on the DASH diet guidelines lowers the level of chemokine C-X-C motif ligand 4 (CXCL4) and regulated upon activation, normal T cell expressed and secreted (RANTES) [22].

The purpose of this study is to assess the effect of the intervention based on the DASH diet model on the MCP-1 plasma concentration, and to assess the possibility of using this chemokine as a marker of changes in the volume and composition of the atherosclerotic plaque.

## 2. Materials and Methods

The dietary intervention to stop coronary atherosclerosis in computed tomography (DISCO-CT) trial enrolled 97 patients in whom coronary artery disease (CAD) was confirmed by computed tomography angiography (CCTA) (NCT02571803) [23]. As per study protocol, all participants were qualified to conservative treatment of CAD; subjects with indications for expanded diagnostics and coronary revascularisation were not included. The remaining inclusion and exclusion criteria were described in detail elsewhere [23]. 92 subjects (5 resigned before completing the study) were randomly allocated to either experimental (optimal medical treatment plus dietary intervention) or control arm (optimal medical treatment alone. A total of 10 blocks of 10 patients were created; in every block, 5 patients were allocated to the experimental and 5 to the control arm. Medical treatment was optimised in both arms according to the guidelines of the European Society of Cardiology (ESC) [24]. Totally, 91 participants completed the trial, and the analysis of atherosclerotic plaque in CCTA was feasible in 89.

This chemokine sub-study enrolled 79 participants of the DISCO-CT trial after additional criteria had been excluded, i.e., diagnosed rheumatoid arthritis (in 11 patients) and a suspicion of liver injury (in 1 patient). Active malignancy was not diagnosed in any of the patients during the trial. The analysis of coronary plaque dynamics described in the Section 3.3 included 77 patients in whom complete imaging data was available.

### 2.1. Dietary Intervention

Dietary counselling was implemented at 6 visits: at the baseline, and after 1, 3, 6, 9 and 12 months. Patients could also benefit from dietary consultations by telephone or email. In the DASH group, basal metabolic rate (BMR) was estimated based on body composition analysis. Together with the assessment of the level of activity, this allowed the establishment of an individual DASH diet plan according to need. In the group of obese patients, the relative to requirements reduction in caloric intake did not exceed 200 kcal and was due to increased physical activity. The energy sources in the nutritional plans were as follows: 52–55% energy from carbohydrates, 16–18% energy from proteins and 30% energy from fats in general.

The assessment of nutrition model included a food frequency questionnaire (FFQ) and a 24 h eating history. Nutrition quality and observance were evaluated in both study arms using the DASH Index. Compliance within 8 critical food groups: cereals, vegetables, fruits, dairy products, meat, nuts/seeds/pips, fats/oils and sweets was assessed. The maximum number of points in each group of foods was ten [25].

### 2.2. Physical Activity

Participants were recommended to increase their physical activity throughout the study regardless of which arm they were allocated to. Recommendations for physical activity were based on ESC guidelines. The following forms of moderately intense physical activity were recommended: Nordic walking, quick marching, swimming, cycling. At each monitoring visit, physical activity data was collected in both study arms. Participants were requested to rank their physical activity as regular (at least 3 sessions of 30 min/week), irregular or none.

### 2.3. Anthropometric Measurements

The measurements of body height and body mass were taken using a stadiometer with a digital scale, InBody BSM 370 (Biospace, South Korea). Body composition analysis was performed twice, at baseline and at follow-up using the InBody S10 analyser (Biospace, South Korea). Total body fat (TBF), total body fat percentage (TBF%), and visceral fat area (VFA) were analysed. Visceral fat area was defined as the intra-abdominal cross-sectional area of visceral adipose tissue (cm^2^). The measurements were performed during fasting and at least 12 h after the last physical activity.

### 2.4. Analysis of Atherosclerotic Plaque

A semiautomated software (QAngioCT version 3.1.3.13, Medis Medical Imaging Systems, Leiden, The Netherlands) was used to analyse the coronary plaque, as previously described [23]. Based on predefined tissue attenuation ranges in Hounsfield units (HU), the following plaque components were distinguished: dense calcium (>351 HU), fibrous plaque (151–350 HU), and non-calcified plaque (−30–150 HU).

Total atheroma volume (TAV), expressed in mm^3^, percent atheroma volume (PAV), expressed in %, and the content of dense calcium, fibrous plaque, and non-calcified plaque, all normalised to TAV and expressed in %, were included in the analysis.

### 2.5. Biochemical Analyses

Blood for biochemical tests was collected from fasting patients at the baseline, after 6 months, and after 12 months of dietary intervention. Plasma MCP-1 concentration was measured using the human CCL2 precoated ELISA (enzyme-linked immunosorbent assay) kit (Biorbyt, Cambridge). The assay was performed according to the manufacturer’s instructions.

### 2.6. Statistical Analysis

The correlations between MCP-1 plasma concentration and dietary factors and between MCP-1 plasma concentration and plaque components were determined with Spearman’s rank test. Means were compared with Student’s *t*-test. Categorical data was compared using Mann–Whitney U test. Differences between groups were compared using the one-way ANOVA test. Linear regression analysis with continuous plaque volume or content as independent variables was performed to investigate the relationship between MCP-1 and plaque dynamics.

The analysis was performed using STATISTICA 13 software with statistical significance set to *p* < 0.05.

## 3. Results

The clinical characteristics of the study groups are presented in Table 1 and Table 2.

The study group demonstrated high level of compliance with the instructions of the DASH eating plan during 12 months of the study. The DASH index in the study group rose from 34.3 ± 14.7 to 60.4 ± 9.15 (*p* = 0.0001). In the control group, this increase was insignificant (from 35.03 ± 13.6 to 38.49 ± 12.3, *p* = 0.138). Table 3 presents a summary of the DASH Index components and daily intake of energy and selected nutrients in both study groups at baseline and after the study completion. The patients also showed changes in their physical activity. The number of patients who declared that they were doing regular physical activity in the DASH group increased from 22 (55%) to 32 (80%) (*p* = 0.004) compared to 9 (23%) to 20 (51%) in the control group (*p* = 0.08). Significant changes in body composition were observed as a result of modified diet habits combined with increased physical activity. In the study group, the total body fat percentage (TBF%) decreased from 33.05 ± 9.06 to 30.1 ± 8.12 (*p* = 0.00015). Conversely, in the control group, the total body fat percentage increased slightly from 32.9 ± 7.34 to 33.7 ± 9.02 (*p* = 0.246). In the DASH group, the visceral fat content was considerably reduced, by 19.9 ± 29.2 cm^2^ (*p* = 0.0001), while in the control group the reduction in the visceral fat content was insignificant (0.18 ± 26.6 cm^2^). In addition, changes in the lipid profile and selected inflammatory markers were analysed. Total cholesterol was reduced by 22.4 mg/dL (*p* = 0.007), LDL cholesterol by 17.89 mg/dL (*p* = 0.008), and hs CRP by 0.12 mg/L (*p* = 0.003). In the control group, the parameters did not change significantly.

### 3.1. Analysis of MCP-1 Plasma Concentration

Baseline level of MCP-1 plasma concentrations in the entire study group was not dependent on high-sensitivity C-reactive protein (CRP), low density lipoprotein (LDL) cholesterol, high-density lipoprotein (HDL) cholesterol, homocysteine or RANTES (Table 4).

**Figure 1:**
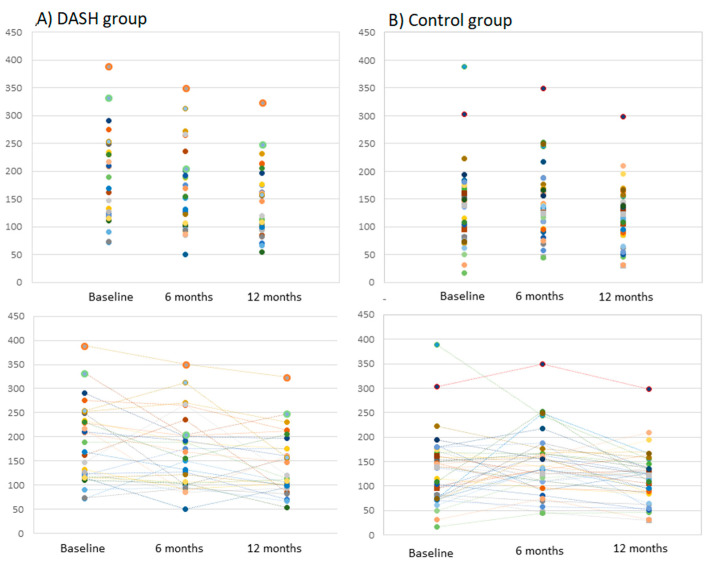
Individual MCP-1 plasma concentrations (pg/mL) and their changes during the study in the DASH group (**A**) and in the control group (**B**).

**Figure 2:**
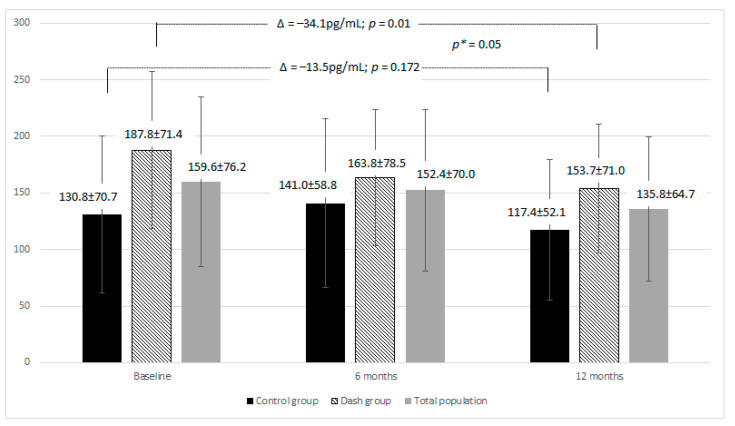
Change in mean plasma MCP-1 concentration (pg/mL) in the whole study group and in subgroups at baseline, and after 6 and 12 months. *p* indicates differences within the group (paired samples *t*-test). p* indicates the difference between the change in the two groups (independent samples *t*-test).

It was not correlated with the visceral fat content either. The baseline MCP-1 plasma concentration in the entire study population was 159.6 ± 76.2 pg/mL and was significantly higher in the DASH group than in the control (*p* = 0.003). Details of baseline MCP-1 levels and changes during follow-up after 6 and 12 months are shown in Figure 1 and Figure 2.

During the study, significant changes in the plasma MCP-1concentration were observed in the DASH group (decrease of 34.1 pg/mL, *p* = 0.01), while in the control group the decrease was not statistically significant (decrease of 13.5 pg/mL, *p* = 0.172). An increase in MCP-1 levels during the study was found in only 17.5% of patients in the DASH group, compared to 35.9% in the control group.

### 3.2. Analysis of a Correlation between MCP-1 and the DASH Index, Selected Dietary Components and Physical Activity

The relationships between baseline and final levels of MCP-1, the total DASH index and DASH Index components were analysed. A significant inverse correlation was found for the vegetable (r = −0.235 *p* = 0.03) and meat intake (r = −0.252, *p* = 0.024) for the entire study population. In addition, correlations were analysed in subgroups: A—the subgroup in which a reduction in MCP-1 levels was achieved throughout the study (N = 58) and B—the subgroup in which an increase in MCP-1 levels occurred (N = 21), showing an inverse correlation of final MCP-1 levels with the vegetable intake (r = −0.286; *p* = 0.0047) in subgroup A. No significant correlations were found in subgroup B. Correlation analysis was also performed for the intake of selected nutrients obtained from the 24 h dietary interview. In the entire study group, a significant inverse correlation was found between baseline MCP-1 level and baseline dietary fibre intake (r = −374; *p* = 0.008). For the final MCP-1 levels, an inverse correlation was shown for folic acid intake (r = −0.234; *p* = 0.038). Analysis in subgroups A and B showed only a correlation in the subgroup A, where MCP-1 inversely correlated with vitamin C intake (r= −0.233; *p* = 0.05).

Second, changes in the baseline-to-follow-up MCP-1 levels were analysed showing no significant correlation with changes in the total DASH Index, both in the whole study population and in the study groups: DASH group and control group. However, a statistically significant inverse correlation was observed for a change in vegetable intake (r = −0.252, *p* = 0.024) and meat intake (r = −0.278, *p* = 0.01) in the entire study population. No significant correlations were observed in the study groups.

**Figure 3:**
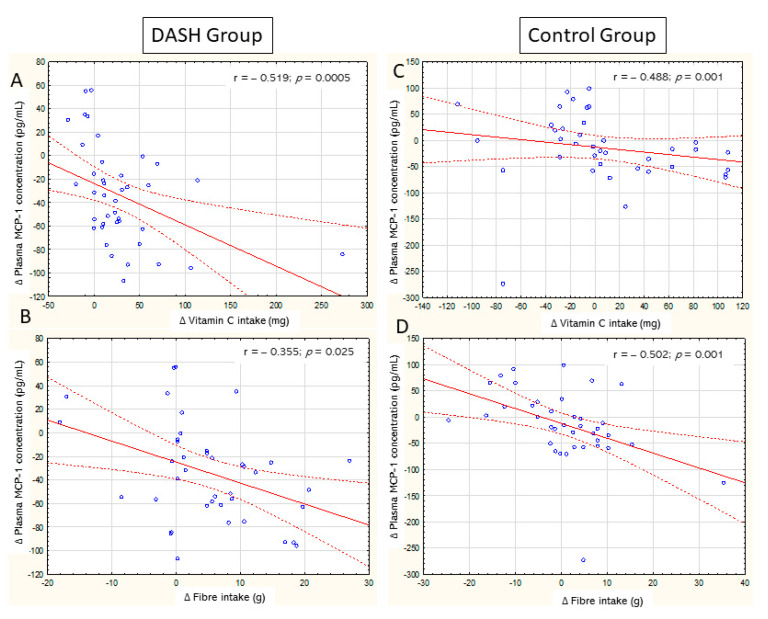
Correlations between the (Δ) change in MCP-1 plasma concentration and the changes in the daily intake of selected nutrients. DASH Group: (**A**) correlation between change in MCP-1 plasma concentration and change in Vitamin C intake; (**B**) correlation between change in MCP-1 plasma concentration and change in fibre intake. Control group: (**C**) correlation between change in MCP-1 plasma concentration and change in Vitamin C intake; (**D**) correlation between change in concentration of MCP-1 and change in fibre intake. Values are mean ± SD.

Analysis of the correlation between changes in MCP-1 plasma concentration and the intake of selected nutrients was performed. Significant positive correlations were shown for changes in the energy intake (r = 0.230, *p* = 0.04), changes in saturated fatty acid intake (r = 0.226, *p* = 0.04), and inverse correlations were shown for fibre intake (r = −0.461, *p* = 0.0002) and vitamin C (r = −0.545, *p* = 0.0001). Correlations were also shown for the same components in the study subgroups: in the DASH group, the r for vitamin C was −0.519 (*p* = 0.0005) and for fibre −0.353 (*p* = 0.025); in the control group, the r for vitamin C was −0.488 (*p* = 0.001), and for fibre −0.502 (*p* = 0.001). Figure 3 shows scatter plots for these correlations. No significant correlations were shown for energy intake (r = 0.206; *p* = 213) and such components as polyunsaturated fatty acids, monounsaturated fatty acids, vitamins of group B, vitamin E, and vitamin A.

The correlation analysis did not show any relationship between physical activity and the change in the MCP-1 in both groups.

### 3.3. Analysis of the Correlation between the MCP-1 Plasma Concentration and Atherosclerotic Plaque Composition

As part of DISCO-CT project, the baseline and final composition of the atherosclerotic plaque was analysed in the study participants based on the coronary computed tomography angiography, whose detailed results were published earlier [23]. A slight increase in plaque volume was observed in the control group, compared to no significant change in the DASH group. A reduction in noncalcified plaque was reported in both the control and the DASH group, while the reduction was significantly higher in the interventional group (*p* < 0.05) [23].

The analysis between baseline and final plaque parameters and MCP-1 plasma concentration showed no significant correlations. No significant correlations were found in subgroups for MCP-1 plasma concentration tertiles, either (Table 5).

Similarly, the analysis between the change in the MCP-1 plasma concentration in the entire study group and the quantitative changes in the plaque did not show significant correlation for any of the parameters analysed (Figure S1 shows scatter plots for plaque components). Therefore, an additional analysis was performed in the study groups. No significant correlations were found in the control group, while in the DASH group a positive correlation was observed between a change in MCP-1 and change in the composition of the calcified component (r = 0.373, *p* = 0.019), and a negative correlation between a change in MCP-1 and change in the composition of non-calcified plaque (r = −0.424, *p* = 0.007). Details are shown in Table 6.

Correlations in the tertiles of PAV changes were also analysed. In the first tertile of PAV changes representing patients with the highest decrease in the atherosclerotic plaque, a significant positive correlation was observed between the change in the MCP-1 level and the change in PAV (r = 0.428, *p* = 0.033) and change in the calcified plaque component (r = 0.468, *p* = 0.018), and a negative correlation was observed between the change in the MCP-1 level and the change in the noncalcified plaque (r = −0.459, *p* = 0.021). No significant correlations were found in the other tertiles.

An additional analysis of changes in the plaque composition was performed for tertiles of changes in MCP-1 for the whole group. For the first tertile of changes in MCP-1 (highest decrease in the MCP-1 level), no significant correlations were observed. However, in the second and third tertile (a slight decrease and increase in MCP-1), there was a statistically significant increase in PAV, an increase in the calcified element and decrease in the noncalcified plaque (Table 7).

MCP-1 levels were also analysed, depending on the tertiles of changes in TAV, PAV, and plaque components. The highest decrease in the noncalcified plaque was observed to be related to a slight increase in MCP-1, while an increase in this element was related to a decrease in the MCP-1. (Table 8).

## 4. Discussion

To the best of our knowledge, this is the first study to assess changes in the level of MCP-1 chemokine after an intervention related to the use of DASH diet in patients with CAD, and the usefulness of this marker in assessing progression or regression of the atherosclerotic plaque.

In earlier studies, we have proved that an intervention based on the DASH diet model causes a decrease in the plasma concentration of RANTES and CXCL4 chemokines [22]. In the present study, we achieved similar results for MCP-1, which decreased by more than 15% in the DASH group, and by 10% in the control group. Baseline MCP-1 plasma concentration in the DASH group was significantly higher than in the control group, which is consistent with previous RANTES and CXCL4 results (in both chemokines the difference was at the limit of statistical significance *p* = 0.05) and indicates a more intense inflammatory state [22]. In contrast to our results, Nygaard et al. did not show any significant effect of the DASH diet on MCP-1 level, but their study included patients with uncontrolled asthma in whom, compared to patients with stable CAD, different pathways of the inflammatory process may prevail [26].

There is evidence in the literature that plasma MCP-1 levels in patients with acute coronary syndrome are significantly higher than in patients with stable coronary artery disease and significantly higher than in healthy subjects [27,28,29]. De Lemos et al. have shown that an increased level of MCP-1 in subjects with acute coronary syndrome, in the fourth quartile (>238 pg/mL, which corresponds to the 90th percentile in the normal healthy population), is correlated with a significantly higher risk of death [30]. The baseline level of MCP-1 in our study was 159.6 pg/mL and was lower than in the Fujita study [31] of patients with stable CAD (240.8 pg/mL) and in the study by Fuchs et al. [32] conducted among patients with manifested CAD and coronary artery stenosis >50% in angiography (609.7 pg/mL). Lower values were shown in the study of Tajfard et. al. with stable CAD, which, for patients with stenosis over 50%, amounted to 124.03 pg/mL, and below 50% to 123.24 pg/mL [33].

The achieved decrease in the MCP-1 level was probably related to a change in lifestyle, especially the introduction of an intensive dietary intervention. Statins have been shown to reduce the MCP-1 concentration [34], but a higher decrease in the DASH group with a comparable percentage of statin therapy use (80% patients in the study group and 84% patients in the control group) indicates an influence of nonpharmacological factors.

The analysis of correlations between dietary components and MCP-1 level (inverse correlation with the vegetable intake, inverse final MCP-1 level correlation with folic acid intake and strong inverse correlations between changes in MCP 1 concentration and changes in fibre and vitamin C intake in both study groups) clearly indicates that the intake of plant-derived products seems to be the most significant factor. The Mediterranean diet, which is rich in fruits, vegetables, whole grain products, nuts and fish, has already shown to reduce the MCP-1 concentration [20,35,36]. High adherence to the Mediterranean diet in these studies was related to a decrease in the number of inflammatory markers, including MCP-1. Conversely, a study conducted among obese women showed that a diet showing low dietary inflammatory index is related to low concentrations of MCP-1 [37]. Interventional studies with the use of the Mediterranean diet analysed the effect of the diet on inflammatory markers during long-lasting interventions, including for 5 years. It appears, however, that an effect may be reached in a shorter time. A short 2-week intervention in healthy women, based on the administration of 500 mL rich in antioxidants vegetable soup, also resulted in a significant decrease in this chemokine [38]. This was related to a significant increase in serum level of vitamin C, which seems to confirm that vitamin C may play an important role in reducing MCP-1. The studies referred appear to confirm that anti-inflammatory diets may modify inflammatory responses, mainly due to the content of antioxidants, fibre and other anti-inflammatory substances, which is consistent with the results of our study. Several studies suggest that folic acid may affect MCP-1 expression and thus contribute to reducing its plasma concentration [39].

The correlation of the DASH Index component with regard to meat intake shown in our study may be related to a reduced intake of oxysterols. A traditional Polish diet involves a high consumption of meat and its products, while the DASH diet allows meat at a limited level. Animal studies show that oxysterols from the diet accelerate destabilisation and rupture of the atherosclerotic plaque, which is related to an increased infiltration/activation of monocytes, MCP-1 expression and matrix metalloproteinase (MMP) activity [40].

Analysis of the baseline and final concentration of MCP-1 in relation to plaque composition did not reveal significant correlations. The results of previous studies are not consistent. In a study by Li et al. of patients with CAD, it was found that the MCP-1 concentration was positively correlated with the percentage of fibrous-fatty plaque and necrotic core, the components that form the noncalcified plaque component in our study and negatively correlated with the percentage of fibrous tissue [41]. Different results were obtained by Fuchs et al., who reported that the MCP-1 concentration was correlated with the coronary plaque burden and that higher MCP-1 levels were related to higher calcium content (r = 0.47, *p* = 0.004), necrotic core (r = 0.38, *p* = 0.02) and lower content of the fibrous tissue (r = −0.34, *p* = 0.03) [30]. Similar results were obtained by Cheng et al., who confirmed that the MCP-1 level was correlated with the coronary plaque burden in patients with unstable coronary artery disease [42]. In the studies by Fuchs et al. and Cheng et al. blood was collected directly from the arterial bed during an interventional procedure, whereas in our study venous blood was collected.

None of these studies assessed if changes in the serial MCP-1 measurements may reflect changes in the atherosclerotic plaque during therapy. The results of our study indicate that an increase in the MCP-1 concentration may be associated with a slight increase in the volume of the atheroma, mainly through an increase in its fibrous and calcified element. This is partially consistent with the results achieved by Fuchs et al. [30]. Surprisingly, our findings suggest an inverse correlation between changes in the MCP-1 concentration and the content of the noncalcified element, related to the highest cardiovascular risk. At the same time, the MCP-1 dynamics best reflects changes occurring in the plaque composition in patients with the highest plaque reduction.

Scarce clinical data, conflicting results and distinct methodology of the published studies do not allow to confirm the utility of MCP-1 as a valid biomarker of coronary plaque dynamics. Changes in circulating MCP-1 may depend on many subtle factors, such as oxidative stress [43], insulin resistance [44] and are probably more dynamic than structural changes observed within the coronary plaque. For these reasons, it seems that although MCP-1 may accurately reflect the effect of antioxidants in the diet, its potential usefulness to assess changes in coronary plaque composition requires further investigation.

## 5. Conclusions

In conclusion, a dietary intervention based on the DASH diet model reduces the concentration of MCP-1, mainly due to an increased intake of plant-derived foods with a high fibre and antioxidant content.

The change in the MCP-1 concentration seems to reflect changes in the coronary atheroma volume and proportions between the calcified and non-calcified plaque elements. Nevertheless, studies on larger groups of patients are necessary to ascertain the feasibility of MCP-1 monitoring as a potential marker of coronary plaque remodelling.

## 6. Limitations of the Study

Patients in the control group received general guidance in accordance with the ESC recommendations for lifestyle changes, which may have influenced their eating behaviours.

Subjects treated with statins were not excluded from the study since statin treatment is considered as first line therapy in patients with CAD.

The intervention to increase physical activity was not fully objectified in this study and relied on patients’ self-reporting during the exercise-oriented interview.

Furthermore, in the study population, atherosclerotic lesions were not advanced and cardiovascular risk was intermediate, which could have affected the dynamics of changes in atherosclerotic plaque and inflammatory markers.

The pilot study design resulted in a small group size, which might have affected the power of the study. To confirm results, it is necessary to conduct further studies on larger groups.

## Figures and Tables

**Table 1:** Selected characteristics of the study populations.

	DASH Group (*n* = 40)	Control Group (*n* = 39)	*p*
Age (years)	59.3 ± 8.14	60.5 ± 7.81	0.226
Sex: men (%)	27 (67)	20 (51)	0.261
Observation time (weeks)	69.4 ± 15.8	64.3 ± 10.7	0.08
Hypercholesterolemia, *n* (%)	40 (100)	38 (97)	0.159
Hypertension, *n* (%)	38 (95)	33 (85)	0.50
Impaired Glucose Tolerance, *n* (%)	3 (7.5)	3 (8)	0.726
Persistent atrial fibrillation, *n* (%)	3 (7.5)	3 (8)	0.938
Chronic kidney disease, *n* (%)	0 (0)	1 (2.5)	0.851
Current smoking, *n* (%)	4 (10)	9 (23)	0.245
Prior smoking, *n* (%)	25 (62)	26 (67)	0.995

Values are mean ± SD or *n* (%). DASH, dietary approaches to stop hypertension.

**Table 2:** Clinical parameters and concomitant medications at baseline and follow-up. Statistically significant *p*-values were marked in bold.

	Baseline				Follow-Up	
	DASH Group (*n* = 40)	Control Group (*n* = 39)	*p*	DASH Group (*n* = 40)	Control Group (*n* = 39)	*p*
Weight (kg)	84.4 ± 16.8	83.0 ± 15.56	0.330	81.6 ± 14.5	81.6 ± 15.9	0.487
Body Mass Index (kg/m^2^)	29.6 ± 4.15	29.09 ± 3.84	0.250	28.6 ± 3.6	28.6 ± 3.9	0.431
Maximal lumen diameter stenosis, %	36.6 ± 11.90	37.7 ± 10.30	0.413	32.2± 10.3	39.0 ± 12.4	**0.011**
Number of lesions >50%	2 (5)	2 (5)	>0.999	2 (5)	6 (15)	0.150
Systolic BP, mmHg	129.4 ± 11.97	129.4 ± 14.78	0.498	125.7 ± 13.6	128.2 ± 13.60	0.214
Diastolic BP, mmHg	79.7 ± 6.58	80.3 ± 8.66	0.368	76.8 ± 9.30	78.23 ± 5.61	0.222
**Medical treatment**						
β-blocker, *n* (%)	21 (52.5)	26 (67)	0.115	22 (55)	27 (69)	0.338
ACE inhibitor, ARB *n* (%)	26 (65)	30 (77)	0.335	24 (60)	29 (74)	0.250
Calcium channel blocker, *n* (%)	13 (32)	10 (25)	0.703	12 (30)	11 (28)	0.703
Diuretic, *n* (%)	10 (25)	16 (41)	0.250	10 (25)	17 (43)	0.179
Number of antihypertensive drugs	1.8 ± 1.10	2.2 ± 1.31	0.090	1.7 ± 1.31	2.4 ± 1.28	**0.046**
Statin, *n* (%)	25 (62)	29 (74)	0.233	32 (80)	33 (84)	0.851
High-intensity dose statin#, *n* (%)	7 (17.5)	7 (18)	0.996	9 (22.5)	8 (20.5)	0.851
Other lipid lowering drugs, *n* (%)	3 (7.5)	2 (5)	0.859	2 (5)	1 (2.5)	0.856
ASA, *n* (%)	23 (57.5)	24 (61)	0.505	26 (65)	29 (74)	0.566
NOAC, *n* (%)	0 (0)	1 (2.5)	0.851	0 (0)	1 (2.5)	0.850
Warfarin, *n* (%)	0 (0)	1 (2.5)	0.851	0 (0)	1 (2.5)	0.851
Clopidogrel, *n* (%)	5 (12.5)	0 (0)	0.338	4 (10)	0 (0)	0.444

Values are mean ± SD or *n* (%). DASH, dietary approaches to stop hypertension; BP, blood pressure; ARB, angiotensin receptor blocker; ACE, angiotensin-converting enzyme; ASA, acetylsalicylic acid; NOAC, new oral anticoagulants. #atorvastatin 40 mg daily or more, rosuvastatin 20 mg daily or more. *p* < 0.05 marked in bold.

**Table 3:** Daily intake of selected nutrients and DASH index values in both study groups. *p* indicates differences within the group (paired samples *t*-test). p* indicates differences between groups (independent samples *t*-test). Statistically significant *p*-values are marked in bold.

	DASH Group *n* = 40		*p*	Control Group *n* = 39		*p*	p*
	Baseline	Follow-Up		Baseline	Follow-Up		
**Daily Nutrients Intake**							
Energy (kcal)	2007 ± 451	1705 ± 446	**0.0002**	1870 ± 650	1905 ± 792	0.415	**0.017**
%fat energy	31.8 ± 6.8	28.3 ± 4.8	**0.005**	31.2 ± 6.7	32.6 ± 8.4	0.262	**0.020**
SAFA (g)	21.0 ± 10.5	18.7 ± 7.0	0.105	21.0 ± 10.9	24.09 ± 8.5	0.08	**0.023**
Vitamin A (µg)	693 ± 559	654 ± 497	0.440	911 ± 1186	666 ± 284	0.107	0.116
Vitamin E (mg)	7.9 ± 4.9	13.8 ± 7.1	**<0.0001**	10.1 ± 5.4	8.9 ± 13.7	0.418	**0.0004**
Folic Acid (µg)	190 ± 75	257 ± 113	**0.0003**	255 ± 160	229 ± 83	0.182	**0.003**
Vitamin C (mg)	72 ± 58	102 ± 70	**0.024**	67 ± 33	74 ± 50	0.233	**0.033**
Dietary fibre (g)	21.4 ± 7.6	28.5 ± 7.7	**0.001**	22.2 ± 9.5	23.1 ± 9.5	0.352	**0.026**
**DASH Index Components**							
Grains	5.4 ± 2.2	8.3 ± 1.6	**<0.0001**	5.30 ± 2.1	5.5 ± 8.2	0.378	**<0.0001**
Vegetables	4.7 ± 1.8	7.2 ± 1.9	**0.0001**	4.65 ± 1.8	5.2 ± 2.0	0.120	**<0.0001**
Fruits	4.2 ± 2.4	6.5 ± 3.0	**0.0001**	4.2 ± 2.4	4.4 ± 2.3	0.319	**<0.0001**
Diary	5.9 ± 2.4	7.6 ± 2.4	**0.001**	5.8 ± 2.5	6.2 ± 2.4	0.242	**<0.0001**
Meat	4.4 ± 3.3	8.9 ± 2.0	**<0.0001**	5.5 ± 3.2	5.2 ± 3.0	0.176	**<0.0001**
Nuts	0.8 ± 2.0	5.4 ± 4.3	**<0.0001**	0.9 ± 2.3	1.75 ± 2.4	0.078	**<0.0001**
Fat	5.9 ± 9.1	9.1 ± 1.5	**<0.0001**	5.9 ± 3.6	6.2 ± 2.4	0.342	**<0.0001**
Sweets	3.3 ± 3.9	7.6 ± 3.0	**<0.0001**	3.4 ± 3.8	3.7 ± 3.8	0.353	**<0.0001**
DASH Index	34.3 ± 14.7	60.5 ± 9.5	**0.0001**	35.0 ± 13.6	38.2 ± 7.6	0.138	**<0.0001**

Values are mean ± SD. DASH, dietary approaches to stop hypertension; SAFA, saturated fatty acids.

**Table 4:** Baseline mean MCP-1 plasma concentrations with respect to age and selected risk factors (*n* = 79). *p* indicates differences between groups (independent samples *t*-test).

Risk Factors		MCP-1 [pg/mL]	*p*
Age (years)	≤60	168.9 ± 81.4	0.121
	>60	150.1 ± 70.3	
hsCRP (mg/L)	≤0.13	165.2 ± 86.1	0.254
	>0.13	153.8 ± 65.1	
Homocysteine (µmol/L)	≤12.1	164.71 ± 60.4	0.286
	>12.1	154.9 ± 88.9	
RANTES (pg/mL)	≤34.4	154.6 ± 79.3	0.279
	>34.4	164.7 ± 73.7	
LDL cholesterol (mg/dL)	≤104.9	166.9 ± 86.7	0.197
	>104.9	152.1 ± 64.1	
HDL cholesterol (mg/dL)	≤56.3	162.4 ± 78.1	0.312
	>56.3	154.0 ± 73.9	
VFA (cm^2^)	≤103.6	165.3 ± 85.4	0.251
	>103.6	153.7 ± 66.1	

Values are mean ± SD. hs-CRP, high-sensitivity C-reactive protein; RANTES, regulated upon activation, normal T cell expressed and secreted; LDL, low density lipoprotein; HDL, high-density lipoprotein; VFA, visceral fat area.

**Table 5:** Analysis of the atherosclerotic plaque composition for tertiles of the MCP-1 plasma concentration in the whole study group. *p*-values were obtained with linear regression analyses with continuous plaque volumes and plaque components’ volumes as independent variables.

	Baseline				Follow-Up			
	MCP-1 Tertile 1 (*n* = 25)	MCP-1 Tertile 2 (*n* = 26)	MCP-1 Tertile 3 (*n* = 26)	*p*	MCP-1 Tertile 1 (*n* = 25)	MCP-1 Tertile 2 (*n* = 26)	MCP-1 Tertile 3 (*n* = 26)	*p*
**Coronary Plaque Volume**								
PAV (%)	34.6 ± 4.9	36.7 ± 6.0	36.9 ± 7.3	0.56	37.9 ± 5.9	35.8 ± 5.9	37.4 ± 6.9	0.79
TAV (mm^3^)	1020.5 ± 91.0	1070.0 ± 355.0	936.4 ± 276.7	0.28	1008.8 ± 300.5	965.5 ± 317.8	1056.6 ± 259.0	0.83
**Coronary Plaque Components**								
Fibrous (%)	47.6 ± 6.0	49.7 ± 5.9	48.3 ± 6.0	0.83	49.0 ± 5.3	49.0 ± 5.6	49.2 ± 5.1	0.66
Dense calcium (%)	10.4 ± 7.5	10.2 ± 5.4	11.8 ± 9.2	0.93	13.3 ± 7.9	15.8 ± 8.0	11.9 ± 8.9	0.71
Noncalcified (%)	42.0 ± 10.5	40.1 ± 7.1	39.9 ± 9.1	0.94	37.7 ± 8.8	35.2 ± 6.7	38.9 ± 9.5	0.84

Values are mean ± SD. PAV, percent atheroma volume; TAV, total atheroma volume.

**Table 6:** Correlation between the change (∆) in MCP-1 plasma concentration and the change in plaque volume and composition in both study groups. Statistically significant *p*-values are marked in bold.

	DASH Group		Control Group	
	r	*p*	r	*p*
∆ PAV (%)	0.181	0.270	0.046	0.785
∆ TAV (mm^3^)	0.211	0.198	0.006	0.972
∆ Fibrous (%)	0.303	0.061	0.102	0.543
∆ Noncalcified (%)	−0.424	**0.007**	−0.034	0.840
∆ Dense calcium (%)	0.373	**0.019**	−0.030	0.856

Abbreviations: PAV, percent atheroma volume; TAV, total atheroma volume.

**Table 7:** Changes (∆) in the volume and composition of the atherosclerotic plaque within tertiles of changes in MCP-1 concentration in the entire study group (*n* = 77). *p*-values were obtained using paired sample *t*-test comparing baseline and follow-up values. p* values were obtained using one-way ANOVA comparing intergroup differences. Statistically significant *p*-values are marked in bold.

	∆MCP-1 Tertile 1 (*n* = 25)	*p*	∆MCP-1 Tertile 2 (*n* = 26)	*p*	∆MCP-1 Tertile 3 (*n* = 26)	*p*	p*
**Coronary Plaque Volume**							
∆ PAV (%)	−0.5 ± 3.7	0.539	1.9 ± 4.1	**0.024**	1.3 ± 3.0	**0.043**	0.060
∆ TAV (mm^3^)	−46.5 ± 159.8	0.159	18.3 ± 163.8	0.574	25.6 ± 134.9	0.342	0.190
**Coronary Plaque Components**							
∆ Fibrous (%)	−1.0 ± 3.1	0.132	1.8 ± 4.3	**0.043**	0.7 ± 3.8	0.376	**0.036**
∆ Dense calcium (%)	1.4 ± 5.2	0.180	4.1 ± 5.9	**0.002**	3.1 ± 4.1	**0.001**	0.175
∆ Noncalcified (%)	−0.5 ± 5.9	0.704	−5.9 ± 8.9	**0.002**	−3.7 ± 5.2	**0.001**	**0.020**

Values are mean ± SD. PAV, percent atheroma volume; TAV, total atheroma volume.

**Table 8:** Changes (∆) in MCP-1 plasma concentration within tertiles of changes in the atherosclerotic plaque volume and composition. P1-2 values represent differences between tertile 1 and tertile 2. P2-3 values represent differences between tertile 2 and tertile 3. P1-3 values represent differences between tertile 1 and tertile 3. P1-2, p2-3, and p1-3 values were obtained using a *t*-test to compare means. p* values were obtained using one-way ANOVA comparing intergroup differences. Statistically significant *p*-values are marked in bold.

		*n*	∆MCP-1 (pg/mL)	*p*_1-2_	*p*_2-3_	*p*_1-3_	p*
**Coronary Plaque Volume**							
**∆PAV**	Tertile 1	25	−24.5 ± 51.8	0.570	0.265	0.540	0.507
	Tertile 2	26	−33.8 ± 64.0				
	Tertile 3	26	−15.4 ± 53.2				
**∆TAV**	Tertile 1	25	−25.5 ± 50.4	0.770	0.760	0.948	0.944
	Tertile 2	26	−21.5 ± 46.7				
	Tertile 3	26	−26.7 ± 71.0				
**Coronary Plaque Components**							
**∆Fibrous**	Tertile 1	25	−43.1 ± 67.3	0.121	0.951	0.087	0.135
	Tertile 2	26	−16.1 ± 54.5				
	Tertile 3	26	−15.3 ± 43.0				
**∆Dense Calcium**	Tertile 1	25	−32.7 ± 48.9	0.339	0.805	0.546	0.667
	Tertile 2	26	−18.6 ± 54.5				
	Tertile 3	26	−22.8 ± 65.6				
**∆Noncalcified**	Tertile 1	25	1.6 ± 45.1	**0.002**	0.280	**0.039**	**0.009**
	Tertile 2	26	−45.8 ± 59.0				
	Tertile 3	26	−28.5 ± 55.6				

Values are mean ± SD. PAV, percent atheroma volume; TAV, total atheroma volume.

## Data Availability

The data presented in this study are available on request from the corresponding authors. The data are not publicly available due to the study is funded by the Institute of Cardiology in Warsaw and management approval is required.

## Supplementary Materials

The following are available online at https://www.mdpi.com/article/10.3390/nu13093047/s1, Figure S1: Scatter plots for the analysis of the relationship between change (Δ) in the MCP-1 plasma concentration and change in plaque volumes and plaque components’ volumes in the whole study group (*n* = 77): A—Δ PAV, B—Δ TAV, C—Δ Fibrous, D—Δ Non-calcified, E—Δ Dense calcium. Solid lines—mean values, dotted lines—2 × standard deviation.
