# Supplementary figures and images for: Usefulness of MCP-1 Chemokine in the Monitoring of Patients with Coronary Artery Disease Subjected to Intensive Dietary Intervention: A Pilot Study
Source: Nutrients. 2021 Aug 30;13(9):3047. doi: 10.3390/nu13093047 (PMC8467171; doi:10.3390/nu13093047)

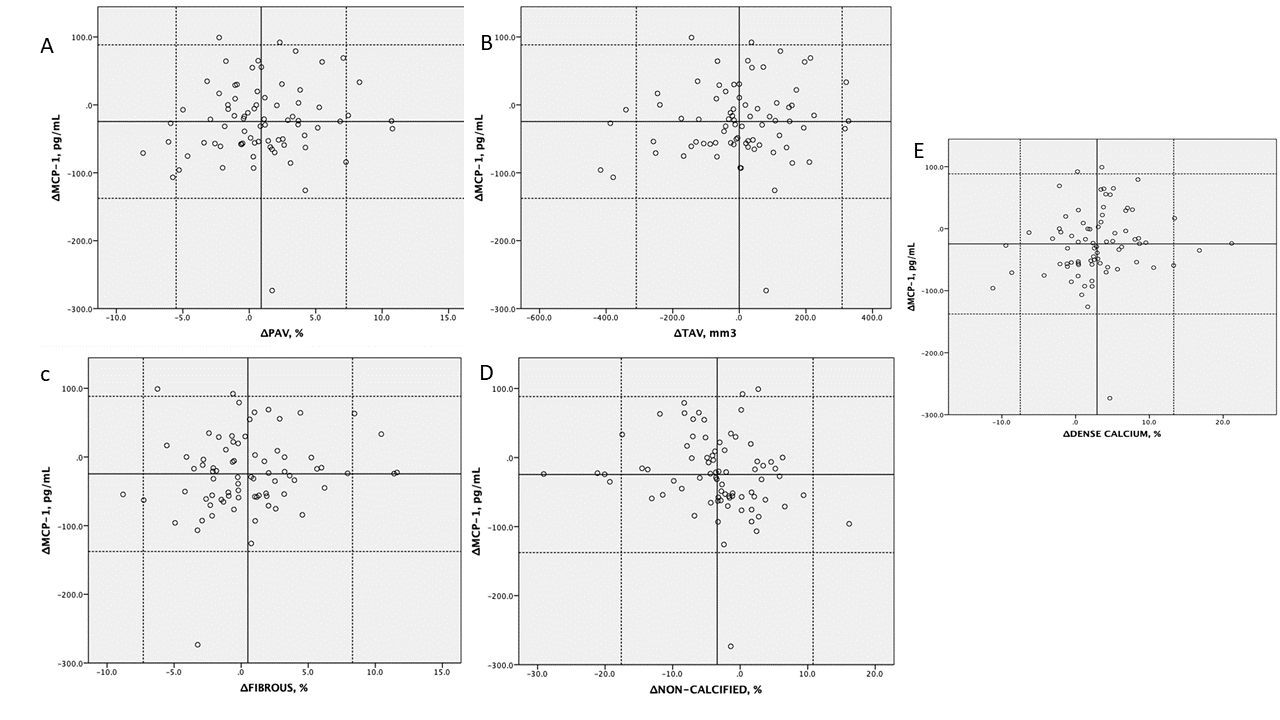

Supplement: Supplementary file 1 [file nutrients-13-03047-s001.zip › nutrients-1285176-supplementary.png]
